# iMeta progress and acknowledgment of reviewers in 2022

**DOI:** 10.1002/imt2.89

**Published:** 2023-02-13

**Authors:** Yong‐Xin Liu, Chun‐Lin Shi, Tengfei Ma, Wubin Ding, Danyi Li, Tong Chen, Jingyuan Fu, Shuang‐Jiang Liu

**Affiliations:** ^1^ Shenzhen Branch, Guangdong Laboratory of Lingnan Modern Agriculture, Genome Analysis Laboratory of the Ministry of Agriculture and Rural Affairs, Agricultural Genomics Institute at Shenzhen Chinese Academy of Agricultural Sciences Shenzhen China; ^2^ ANGENOVO Viken Norway; ^3^ Centre for Grassland Microbiome, State Key Laboratory of Grassland Agro‐ecosystems, College of Pastoral Agricultural Science and Technology Lanzhou University Lanzhou China; ^4^ Center for Computational and Genomic Medicine The Children's Hospital of Philadelphia Philadelphia Pennsylvania USA; ^5^ R Institute Co. Ltd. Beijing China; ^6^ State Key Laboratory Breeding Base of Dao‐di Herbs, National Resource Center for Chinese Materia Medica China Academy of Chinese Medical Sciences Beijing China; ^7^ Department of Genetics, University Medical Center Groningen University of Groningen Groningen The Netherlands; ^8^ State Key Laboratory of Microbial Resources, Institute of Microbiology Chinese Academy of Sciences Beijing China; ^9^ State Key Laboratory of Microbial Technology Shandong University Qingdao China

## Abstract

Milestones of the first year of iMeta. iMeta is an open‐access Wiley partner journal launched by iMeta Science Society consisting of worldwide scientists in bioinformatics and metagenomics. In 2022, iMeta released four issues, including 60 publications with a total of 340 citations. iMeta has been indexed in several databases, including Google Scholar, Crossref, CNKI, Dimensions, PubMed (partial), DOAJ, and Scopus. Thanks to the editorial board members and reviewers for their contributions to the iMeta in 2022.

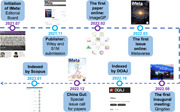

“***iMeta***” is an open‐access Wiley partner journal launched by iMeta Science Society consisting of worldwide scientists in bioinformatics and metagenomics. iMeta aims to promote microbiome and bioinformatics research by publishing original research, methods/protocols, and reviews. The goal is to publish high‐quality papers (top 10%) targeting a broad audience. Unique features include video submission, reproducible analysis, figure polishing, APC waiver, and promotion by social media with 500,000 followers [1]. In 2022, iMeta released four issues (Figure 1), including 60 publications with a total of 340 citations (https://app.dimensions.ai/ by December 31, 2022). The top three highly cited papers were ImageGP [2], Majorbio Cloud [3], and Sangerbox [4], with more than 50 citations, respectively.

**Figure 1:**
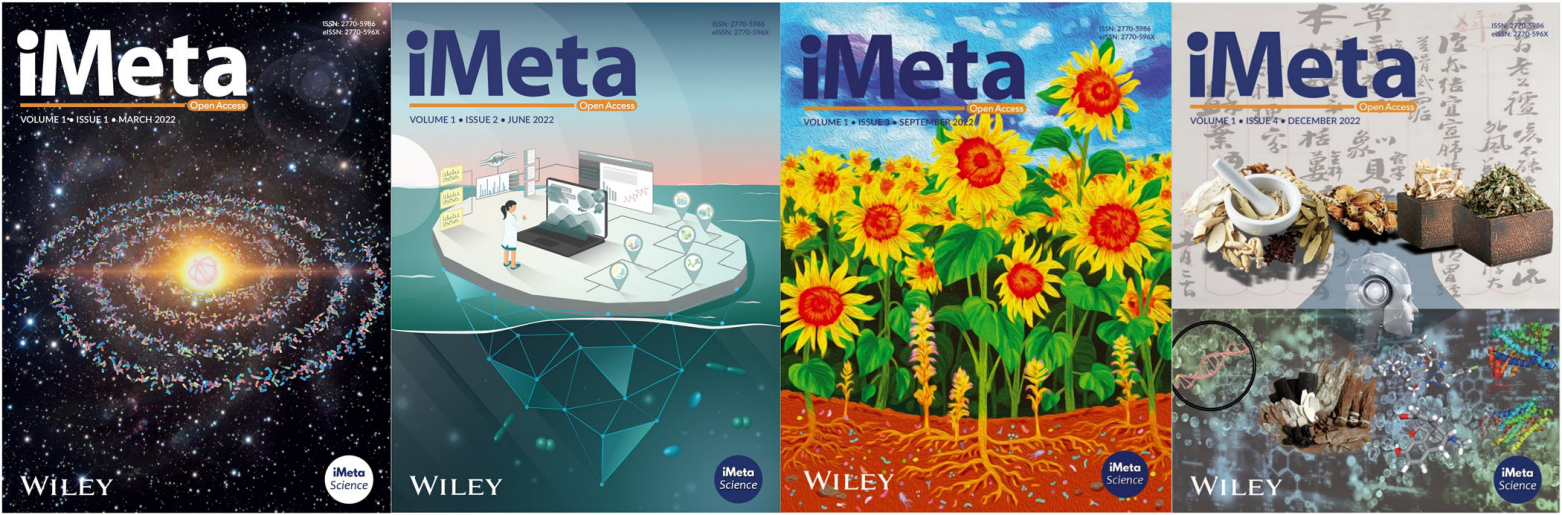
Brief introduction of covers from 2022 issues. Issue 1: The galaxy represents the complexity and value of bioinformatics and metagenomics. DNA, which represents genetic components that guide biological diversity, is at the center of the galaxy. The spiral arms are the microbiome welcoming scientists from all over the world to make novel discoveries. Let us usher in the metaverse era of the microbiome. Issue 2: iMetaLab, a metaproteomics platform from Daniel Figeys' group. The figure represents the visualization of big data mining from the ocean of microbiomes. The implication is that various omics in microbiome research can develop useful and easy‐to‐use analysis tools, and create high‐quality analysis platforms as an infrastructure. Issue 3: Sunflower Orobanche Microbiome, sunflower‐ microbe interaction in the prevention and control of Orobanche parasitism by a team of Academicians James M. Tiedje and Yanbing Lin. The style of painting pays homage to Van Gogh's sunflower, the masterpiece of Impressionism, and research on plant microbiome, which represents vitality, flourish, and the sustainable development of agriculture. Issue 4: TCM‐Suite, a digital analysis platform for traditional Chinese medicine identification and pharmacology network research from Kang Ning's group. The cover reflects the collision and integration of traditional Chinese medicine knowledge and modern omics data under the blessing of artificial intelligence and represents the new vitality of traditional Chinese medicine with the help of modern technologies.

Since the first paper was published in February and the first issue was released on March 2022, publications of iMeta have been indexed in several databases, including Google Scholar https://scholar.google.com/citations?user=u181x38AAAAJ), Crossref (https://search.crossref.org/?q=imeta&from_ui=yes), CNKI (https://www.cnki.net/), Dimensions (https://app.dimensions.ai/discover/publication?and_facet_source_title=jour.1412973), PubMed (partial, https://pubmed.ncbi.nlm.nih.gov/?term=%222770-596X%22), DOAJ (https://doaj.org/toc/2770-596X), and Scopus (https://suggestor.step.scopus.com/progressTracker/?trackingID=E44A9E02E9092B0D) (Figure 2A).

**Figure 2:**
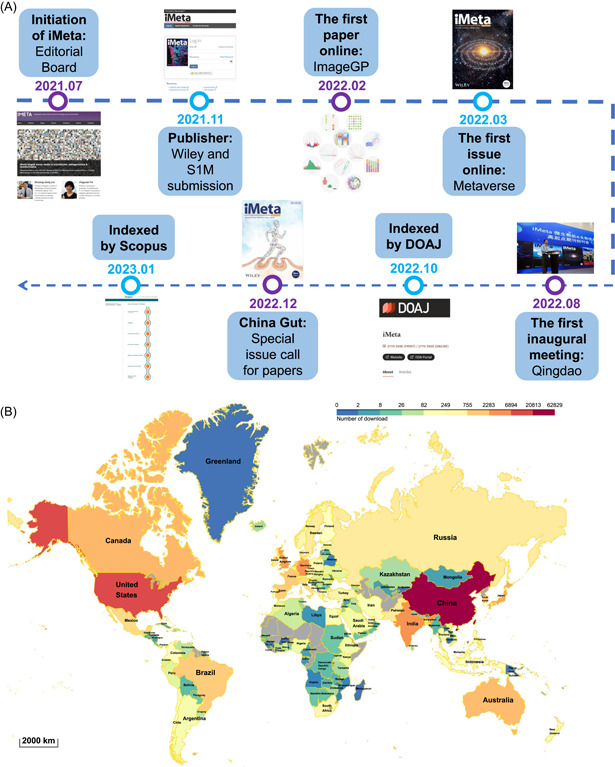
Key events and globe audience distributions of iMeta in 2022. (A) iMeta was initiated in July 2021, collaborated with Wiley in November 2021, and followed by the first paper and first issue online in February and March 2022, respectively. The first inaugural meeting was successfully held at Qingdao in August 2022, and a special issue is currently available related to the China Gut Conference 2023. By January 2023, publications of iMeta have been fully indexed by DOAJ and Scopus. (B) The readers of iMeta are mainly from China (including Hongkong, Taiwan, and Macao), USA, Germany, India, UK, Japan, Canada, Australia, South Korea, Netherlands, France, Singapore, Brazil, Spain, Mexico, Italy, Denmark, Switzerland, Russia, Sweden, Ireland, Pakistan, Israel, Belgium, South Africa, Thailand, Turkey, Poland, Norway, Chile, Colombia, Austria, Philippines, Finland, and Portugal.

According to Wiley (https://insights.wiley.com/), publications of iMeta display board interest of audiences, attracting 115,809 downloads from 165 countries and regions in 2022 (Figure 2B, Supporting Information: Table S1). Notably, a single publication “Complex heatmap visualization” [5] has been downloaded 11,537 times in 2022 (Supporting Information: Table S2).

The editorial board of iMeta currently contains around 1000 members from 29 countries and regions. With the advantages of such a wonderful group, iMeta can make the first decision on average 25 days after submission, providing responses from five reviewers or more. The Editors‐in‐Chief, executive editors, and staff of iMeta are very grateful to the following editorial board members and scientists who dedicated their considerable time and expertise to the journal by serving as reviewers by December 31, 2022 (Table 1, 382 reviewers completed 685 reviews).

**Table 1:** **List of iMeta reviewers in 2022**.

Guy R. Adami	Newell W. Johnson	Paramesh Venkatesh
Sam Al‐Dalali	Feng Ju	Luis Vitetta
Samia Almoughrabie	Kohmei Kadowaki	Jenifer B. Walke
Busayo Joshua Babalola	Emmanouil Karteris	Hu Wan
Gude Baoer	Weidong Kong	Haitao Wang
Zarrin Basharat	Konstantinos Kormas	Hongwei Wang
Maurizio Battino	Paul Henning Krogh	Jianjun Wang
Johan Bengtsson‐Palme	Leo Lahti	Jinfeng Wang
Punyasloke Bhadury	Daoliang Lan	Jinhua Wang
Guillaume J. Bilodeau	Gabriele R. Lara	Jun Wang
Surjyo Jyoti Biswas	Xinggen Lei	Kai Wang
Jacob W. Bledsoe	Bin Li	Lei Wang
Didier Bouchon	Guanjian Li	Liang Wang
John P. Bowman	Guoliang Li	Miaoxiao Wang
Felix Broecker	Hongjin Li	Mingbang Wang
Feng Cai	Houkai Li	Qingshi Wang
Jiabao Cao	Huan Li	Shaolin Wang
Yan Cao	Jing Li	Wei Wang
Yunpeng Cao	Kun Li	Weijie Wang
Franck Carbonero	Min Li	Yiming Wang
Massimiliano Cardinale	Pengsong Li	Yuhao Wang
Sankha S. Chakrabarti	Rong Li	Yulin Wang
You Che	Ruilin Li	Zeneng Wang
An‐Tian Chen	Shun Li	Zhang Wang
Guoan Chen	Tongtong Li	Zhi Wang
Hanqing Chen	Weiguang Li	Xiaoman Wei
Liang Chen, CAS	Wenjun Li	Yan Wei
Liang Chen, Jilin University	Wenxuan Li	Xie Weijie
Lianmin Chen	Yaoming Li	Bryan Wong
Qinglin Chen	Yong Li	Hongjing Wu
Tong Chen	Yuan Li	Linkun Wu
Wei Chen	Bin Liang	Shengru Wu
Wei‐Hua Chen	Yantao Liang	Xingqiang Wu
Xingjian Chen	Yuting Liang	Yibo Wu
Zhangran Chen	Chen Liao	Yaoyao Xia
Liang Cheng	Wenfei Liao	Zhi‐Chao Xia
Mingyue Cheng	Yu‐Chieh Liao	Jindong Xie
Jie Cui	Hao Lin	Liwei Xie
Lei Dai	Huang Lin	Yi Xiong
Tianjiao Dai	Rui Lin	Hongzhi Xu
Valeria D'Argenio	Yongxin Lin	Jia Xu
Surajit Das	Leng Ling	Jin Xu
Bonald C. de Figueiredo	Zongxin Ling	Jun Xu
Nadieh de Jonge	Chang Liu	Sai Xu
Fei Deng	Chao Liu	Yungang Xu
Jinhai Deng	Jinxin Liu	Ling Xu
Ye Deng	Lianliang Liu	Ran Xue
Yiqin Deng	Qingfu Liu	Yi Yan
Paltu Dhal	Songlin Liu	Fenglong Yang
Wei Ding	Yang‐Yu Liu	Gaowen Yang
Li‐Na Dong	Yingzhi Liu	Jialiang Yang
Bingyao Du	Yong‐Xin Liu	Jianxia Yang
Hongzhi Du	Antonio Lopez	Jun Yang
Tim Dumonceaux	Ruben Lopez‐Mondejar	Teng Yang
Alexander Eiler	Bo Lu	Yang Yang
Yong Fan	Hongye Lu	Yi Yang
Mingliang Fang	Xiao Luo	Yuchun Yang
Wensheng Fang	Zhiwen Luo	Yuyi Yang
Zhencheng Fang	Xu‐Cong Lv	Yuzhan Yang
Karoline Faust	Bin Ma	Zhikai Yang
Kai Feng	Jing Ma	Wen Yao
Youzhi Feng	Ka‐Wai Ma	Zhiyuan Yao
Serguei Fetissov	Tengfei Ma	Mao Ye
Marcello Fiorani	Xi Ma	Chengliang Yin
Luiz M. R. Gadelha	Yingke Ma	Yanbin Yin
Jorge Galindo‐Villegas	Elizabeth K. Mallott	Guangchuang Yu
Cheng Gao	Balachandran Manavalan	Hang Yu
Feng Gao	Peter Manning	Huichuan Yu
Yi‐Zhou Gao	Xia Mao	Ke Yu
Yuan Gao	Krishna Prahlad Maremanda	Ollie Yiru Yu
Zheng Gao	John G. McMullen	Bao‐Wen Yuan
Maria Gazouli	Sushil Middha	Jing Yuan
Xuejun Ge	Yogendra Nayak	Jun Yuan
Arthur R. Gilmour	Bruce Ni	Zuo‐Fei Yuan
Amit Goel	Yan Ni	Chen Yue
Renjun Gu	Ivan Nikolić	Suling Zeng
Zuguang Gu	Henrik R. Nilsson	Qixiao Zhai
Paulo Ivonir Gubiani	Ben Niu	Chun‐Hong Zhang
Audrey Gueniche	Jan Krzysztof Nowak	Dan Zhang
Liang Guo	Jingjing Peng	Fang Zhang
Linjie Guo	Wei Peng	Jianchao Zhang
Weilong Guo	Xian Peng	Jingying Zhang
Chirag Gupta	Luciano Pinotti	Junya Zhang
Jinming Han	Elliott Price	Lei Zhang
Yong‐He Han	Meng Pu	Liang Zhang
Xiuli Hao	Zhen Qin	Li‐Mei Zhang
Zulfiqar Hasan	Mei Ran	Lin Zhang
Kenji Hashimoto	Lijuan Ren	Meiling Zhang
Guo‐Qing He	Kunal Roy	Minliang Zhang
Jianrong He	Zengliang Ruan	Weipeng Zhang
Jing He	Tongling Shan	Wen Zhang
Jun He	Weitao Shen	Wenjing Zhang
Xiaoqing He	Xiaotao Shen	Xiaoning Zhang
Xuesong He	Yingbo Shen	Xin Zhang
Yan He	Yizhi Sheng	Xingxu Zhang
Zhili He	Jian‐Yu Shi	Yang Zhang
Zilong He	Mang Shi	Yun Zhang
Mingsheng Hong	Wenyu Shi	Yunzeng Zhang
Anyi Hu	Xiaojian Shi	Zhenggui Zhang
Fang Hu	Yu Shi	Fangqing Zhao
Shengwei Hu	Watshara Shoombuatong	Fazhu Zhao
Weigang Hu	Chunxu Song	Jinxin Zhao
Xiaofei Hu	Gang Song	Xue Qiang Zhao
Xiaoting Hua	Jiangning Song	Yuxiang Zhao
Hai‐Jian Huang	Zehe Song	Guoliang Zheng
Jiayuan Huang	Renan Souza	Haizhen Zheng
Jinyan Huang	Christopher Staley	Jusheng Zheng
Jumin Huang	Mikael Strube	Wei Zheng
Shi Huang	Jiacan Su	Ying Zheng
Shimeng Huang	Shibing Su	Yue Zheng
Yuan Huang	Xiaoquan Su	Ziqiang Zheng
Wei‐Lun Hung	Chengcao Sun	Zhenhui Zhong
Waqar Hussain	Haixi Sun	Fangyuan Zhou
Amanul Islam	Junming Sun	Hong‐Wei Zhou
William W. Ja	Yang Sun	Jian‐Guo Zhou
Steven M. Jay	Yanni Sun	Xin Zhou
Che Ok Jeon	Zhihong Sun	You Zhou
Baolei Jia	Zhoutong Sun	Youlang Zhou
Chao Jiang	Kazuki Suzuki	Zhigang Zhou
Jing‐Zhe Jiang	Naoko Takezaki	Ce Zhu
Mingkai Jiang	Song Tang	Guoxing Zhu
Shangtao Jiang	Wenjing Tang	Ying Zhu
Xu Jiang	Xiangming Tang	Jiye Zhu
Jinzhen Jiao	Yayuan Tang	Xin Zong
Shuo Jiao	Wen Tao	Quan Zou
Yang Jiao	Giovanni Tarantino	Tao Zuo
Wenyi Jin	Iman Tavassoly	
Gongchao Jing	Ivone Vaz‐Moreira	

*Note*: Sorted by the last name, 382 reviewers completed 685 reviews.

Additionally, iMeta is recruiting Youth Editorial Board Members (https://onlinelibrary.wiley.com/page/journal/2770596x/homepage/youth-ebm-recruitment).

## AUTHOR CONTRIBUTIONS

Yong‐Xin Liu and Chun‐Lin Shi drafted the paper. Tengfei Ma and Wubin Ding proceeded with the data analysis and visualization. All authors revised and approved the final manuscript.

## CONFLICT OF INTEREST STATEMENT

Shuang‐Jiang Liu and Jingyuan Fu are the Editors‐in‐Chief of iMeta. Yong‐Xin Liu, Tong Chen, and Danyi Li are the executive editors of iMeta. Chun‐Lin Shi and Tengfei Ma are the academic editors of iMeta. The authors have declared no competing interests.

## Supporting information

Supporting information.

## Data Availability

The data and scripts can be found at GitHub https://github.com/iMetaScience/iMeta. All data of the figures can be downloaded in GitHub or Supporting Information: tables. Supporting Information: materials (figures, tables, scripts, graphical abstract, slides, videos, Chinese translated version, and updated materials) may be found in the online DOI or iMeta Science https://www.imeta.science/.
